# Role of *IscA1* in Low-Frequency Magnetic Field-Associated Changes in *Monascus* Pigments and Citrinin Levels in *Monascus purpureus*

**DOI:** 10.3390/jof12080585

**Published:** 2026-08-07

**Authors:** Donghui Li, Yang Yang, Jialan Zhang, Li Li, Yingbao Liu, Suo Chen, Mengxiang Gao

**Affiliations:** 1 College of Life Science, Yangtze University, Jingzhou 434025, China; 2024710973@yangtzeu.edu.cn (D.L.); 2023721066@yangtzeu.edu.cn (Y.Y.); lily2012@yangtzeu.edu.cn (L.L.); liuyingbao@yangtzeu.edu.cn (Y.L.); chensuo9803@126.com (S.C.); 2 College of Animal Science and Technology, Yangtze University, Jingzhou 434025, China; zhangjl@yangtzeu.edu.cn; 3 Institute of Food Science and Technology, Yangtze University, Jingzhou 434025, China

**Keywords:** *Monascus purpureus*, *IscA1* gene expression, low-frequency magnetic field, intracellular iron distribution, *Monascus* pigments, citrinin

## Abstract

We constructed *IscA1* overexpression (*IscA1*^+^) and RNA interference (RNAi-*IscA1*) strains to investigate the role of *IscA1* in regulating the synthesis of *Monascus* pigments (MPs) and citrinin (CIT) in *Monascus purpureus* under low-frequency magnetic field (LF-MF) exposure. Both *IscA1*^+^ and RNAi-*IscA1* strains exhibited altered nutritional growth and sexual reproduction; however, asexual reproduction, the ultrastructure of spores and mycelia, and biomass in liquid fermentation, remained unaffected. The production of yellow and orange pigments increased in the *IscA1*^+^ strain, while the production of red pigments decreased in the RNAi-*IscA1* strain. The expression level of the *IscA1* was shown to determine intracellular iron ion content in *M. purpureus*. Exposure to 1.6 mT LF-MF did not impact biomass but significantly enhanced MPs and decreased intracellular iron content on the 6th day of fermentation in both *IscA1*^+^ and RNAi-*IscA1* strains, while increasing CIT production in the *IscA1*^+^ strain. The interplay between *IscA1* gene expression and LF-MF exposure jointly regulates the expression of genes related to iron–sulfur cluster assembly, iron homeostasis, and the synthesis of MPs and CIT. These results suggest that LF-MF enhances the expression of the *IscA1* gene in *M. purpureus*, promotes iron–sulfur cluster assembly, regulates intracellular iron distribution, and influences the synthesis of MPs and CIT.

## 1. Introduction

*Monascus* pigments (MPs) are beneficial secondary metabolites synthesized by *Monascus* spp. These pigments are predominantly categorized as yellow pigment (YP), orange pigment (OP) and red pigment (RP). MPs exhibit various physiological activities, including anticancer properties, and the ability to lower blood sugar and lipid levels [1]. In recent years, MPs have emerged as the fastest-growing natural food colorant in China, finding widespread application in the food, cosmetics, and pharmaceutical industries [2]. However, the fermentation of *Monascus* for MP production also generates citrinin (CIT), which targets the kidneys, leading to enlargement and potentially resulting in renal failure [3]. Consequently, a crucial objective in MP production is to effectively control CIT production while simultaneously increasing the production of MPs.

The low-frequency magnetic field (LF-MF) exerts a significant promoting effect on the synthesis of monacolin K, ergosterol, and γ-aminobutyric acid in *M. purpureus* [4,5]. An LF-MF of 50 Hz and 0.1 mT does not impact the mycelial growth of *M. purpureus*, while an intensity of 0.4 mT significantly promotes growth [6]. At this intensity, the production of RP and YP during the solid fermentation of *M. purpureus* increased by 59% and 65%, respectively, compared to the control group [7]. Moreover, at a LF-MF intensity of 1.6 mT, a noticeable inhibition of CIT production is observed during the liquid fermentation of *M. purpureus*, resulting in an increase in the production of MPs [8]. To investigate the underlying mechanisms, researchers have examined the generation of reactive oxygen species (ROS), the elimination of free radicals, the activity of antioxidant enzymes (catalase, superoxide dismutases, glutathione S-transferase), and the expression of relevant genes and proteins to elucidate how LF-MFs suppress CIT production and enhance MPs synthesis [9]. However, these findings indicate only a correlation between the regulation of MPs and CIT synthesis by LF-MFs and specific physiological indicators. They do not elucidate specific action points of the effects produced by LF-MFs or the physiological processes they trigger.

The A-type iron–sulfur cluster protein (IscA) serves as a scaffold protein that is essential for the assembly of iron–sulfur clusters [10]. Furthermore, IscA1 has been identified as a magnetoreceptor, termed MagR, in animal species [11]. Research indicates that IscA is widely distributed among diverse species, exhibiting diverse distribution patterns, while the functions of IscA proteins are highly conserved across these species [12]. Additionally, IscA functions as the iron center of the iron–sulfur cluster assembly protein and serves as an iron-binding protein responsible for the assembly and delivery of [2Fe-2S] and [4Fe-4S] clusters [13]. IscA participates in several physiological processes, including iron homeostasis, mitochondrial function, energy metabolism, hypoxic response, and cell fate determination [14,15,16]. Bioinformatics analysis has identified a protein in *M. purpureus* that is highly homologous to IscA (IscA1/MagR) [17], specifically the IscA1 protein in the iron–sulfur cluster synthesis.

The role of *IscA1* in regulating the synthesis of MPs and CIT under LF-MF exposure remains unclear. To address this, we constructed *IscA1* RNA interference (RNAi-*IscA1*) and overexpression strains (*IscA1*^+^) strains. We examined mycelial and cellular morphology and compared the effects of magnetic field exposure on the production of MPs and CIT, the intracellular and extracellular iron content, as well as the relative expression levels (RELs) of genes involved in the iron–sulfur cluster assembly system (*IscA1*, *IscS*, and *IscU*), iron homeostasis (glutaredoxin *GrxD5*, transcription factors *HapX* and *SreA*), MPs synthesis regulatory (*pksPT* and *pigR*), and CIT synthesis regulatory (*pksCT* and *ctnR*) . This comprehensive analysis aims to investigate the role of *IscA1* in regulating of MPs and CIT synthesis under LF-MF exposure.

## 2. Materials and Methods

### 2.1. Strains Culture and Growth Conditions

The strain *M. purpureus* (CCTCC AF 2020020, Culture Collection of China Center for Type Culture Collection, Wuhan, China) stored in our laboratory [18] was utilized to create both RNAi-*IscA1* and *IscA1*^+^ strains. The preservation and cultivation of *M. purpureus* followed the methodologies described in the literature [19]. Briefly, the strains were preserved on potato dextrose agar (PDA) slants at 4 °C and subsequently cultured on Czapek yeast extract agar (CYA) for sporulation at 30 °C over a period of 7 days. Spores were harvested using 10 mL of sterile water and quantified with a hemocytometer. Spores from different strains were inoculated at a concentration of 2.5 × 10^5^ spores/mL by adding 2 μL to PDA/CYA medium, which were then incubate inverted at 30 °C in the dark. For liquid fermentation, a final concentration of 5 × 10^3^ spores/mL was inoculated into 18 × 180 mm test tube containing 10 mL of potato dextrose broth (PDB) and incubated at 30 °C with shaking at 200 rpm. Liquid-state fermentation was evaluated for biomass and secondary metabolites production, as well as for genomic DNA and RNA extraction. The hyphae on the PDA medium were examined for colony morphology, cleistothecium characteristics, and spore morphology. Luria–Bertani (LB) medium containing kanamycin (50 μg/mL) or ampicillin (100 μg/mL) was used to culture *Escherichia coli* 5α and *Agrobacterium tumefaciens* EHA105 (WEIDI, Shanghai, China) at 37 °C for vector construction or transformation.

### 2.2. LF-MF Exposure

PDB-inoculated spores were exposed to LF-MF (frequency 50 Hz) with intensities of 0, 0.9, 1.6, and 2.0 mT for 2 days at 30 °C, respectively. The design and specifications of the LF-MF apparatus used in this experiment were described in detail by Zhang et al. [4]. Following this exposure, the mixture was cultivated at 30 °C and 200 rpm for 4 days, during which biomass, and MPs production were quantified. Furthermore, after LF-MF exposure, the mixture was cultivated under the same conditions to assess CIT production as well as both intracellular and extracellular iron content. In experiments measuring iron ions, preventing iron contamination is crucial. This can be achieved by utilizing ultrapure water for the preparation of culture media and reagents, as well as employing iron-free utensils.

### 2.3. Construction of IscA1 RNA Interference and Overexpression Strain

#### 2.3.1. Construction of *IscA1* RNA Interference Strain

RNAi-*IscA1* was constructed using a dual promoter strategy combined with *Agrobacterium*-mediated transformation (AMT) [20]. Plasmids were isolated using a plasmid extraction kit, while total RNA was extracted from the mycelia of *M. purpureus* with Trizol reagent. Reverse transcription was performed using the HiScript IQ RT SuperMix for complementary DNA (cDNA) following the manufacturer’s protocol. All reagents were obtained from Vazyme Biotech Co., Ltd. (Nanjing, China). The gpdA promoter was sequentially amplified from the plasmid pBARGPE1-Hygro-EGFP (Miaoling Plasmid, Wuhan, China), while the coding sequence (CDS) of the *IscA1* gene was amplified from *M. purpureus* cDNA, and the trpC promoter was amplified from the plasmid pSKH. Following purification of the PCR products with a DNA purification kit, the RNA interference cassette was created through fusion PCR. Both the pCAMBIA3300 plasmid and the RNA interference cassette were double-digested with *EcoR*I and *Xba*I. Following purification of the digestion products, the RNA interference cassette was ligated into the digested pCAMBIA3300 plasmid to create pCAMBIA3300+ RNAi using T4 ligase. Subsequently, both the pKN1 and pCAMBIA3300+ RNAi plasmids were digested with the XbaI restriction enzyme; the *NeoR* fragment excised from the pKN1 plasmid was ligated into the digested pCAMBIA3300+ RNAi plasmid, resulting in the construction of the pCAMBIA-RNAi-R vector [19], which serves as the RNA interference vector (Supplementary Figure S1). The RNA interference vector was integrated into the *M. purpureus* genome via AMT. The primer sequences utilized to construct the RNAi-*IscA1* strain are presented in the Supplementary Table S1.

#### 2.3.2. Construction of *IscA1* Overexpression Strain

The *IscA1*^+^ strain was constructed through homologous recombination, fusion PCR, and AMT methodologies. Briefly, genomic DNA of *M. purpureus* was extracted from the mycelia of *M. purpureus* using the cetyltrimethylammonium bromide (CTAB) method [21]. This genomic DNA served as a template for the PCR amplification of the left and right homologous arms of the *IscA1* gene. The gpdA promoter and the trpC terminator were amplified from pBARGPE1-Hygro-EGFP, while the CDS of the *IscA1* gene was amplified from *M. purpureus* cDNA. These three fragments are integrated to create the expression cassette (PgpdA-CDS-TtrpC) via fusion PCR [22]. Additionally, the *NeoR* resistance gene was amplified from the plasmid pKN1. The fusion PCR method was implemented to sequentially assemble the left homologous arm of the *IscA1* gene, the overexpression cassette, the *NeoR* gene, and the right homologous arm of the *IscA1* gene, leading to the formation of homologous recombination cassette. This cassette, together with the pCAMBIA3300 plasmid, was subsequently double-digested with XhoI and XbaI, and then ligated using T4 ligase to construct the *IscA1* overexpression vector (Supplementary Figure S2). Following established protocols, *A. tumefaciens* EHA105 was employed to infect wild-type *M. purpureus*, facilitating targeted gene replacement. The primer sequences used to construct the *IscA1*^+^ strain are provided in Supplementary Table S2.

#### 2.3.3. Verification of RNAi-*IscA1* and *IscA1*^+^ Strain

The verification of the constructed recombinant plasmid, the electroporated *Agrobacterium*, and the transformants of *M. purpureus* were respectively conducted. The constructed recombinant plasmid and the electroporated *Agrobacterium* were validated using antibiotic selection media and colony PCR. The transformants of *M. purpureus* grown on G418 selection media underwent DNA extraction using microwave methods [23] for PCR verification, thereby reducing the incidence of false positive transformants. Subsequently, genomic DNA from positive transformants was extracted via the CTAB method for targeted verification, resulting in the identification of site-directed mutation strains. The RELs of wild type (WT), RNAi-*IscA1* and *IscA1*^+^ strains were analyzed using real-time quantitative PCR (RT-qPCR) experiments.

### 2.4. Observation of Colony Morphology, Cleistothecium Characteristics, and Spore Morphology

#### 2.4.1. Observation Using an Optical Microscope

Colony morphology on the PDA medium was examined every 24 h through photographic documentation, and the colony diameter was measured using a Vernier caliper. Hyphal samples were prepared from PDA/CYA medium employing the slice method, allowing for the observation of cleistothecia characteristics, and spore morphology of *M. purpureus* under an upright microscope (BX63, Olympus, Tokyo, Japan) equipped with a DP73 digital camera.

#### 2.4.2. Observation Using a Scanning Electron Microscopy

Samples for scanning electron microscopy (SEM) were prepared according to established protocols [24], with some modifications. Cells cultivated on day 8 PDA/CYA medium were fixed overnight at 4 °C using a 2.5% (*w*/*v*) glutaraldehyde buffer (pH 7.2). Following fixation, the cells were washed with 0.1 M phosphate buffer solution (PBS) (pH 7.4). The cleaned cells were sequentially dehydrated with increasing concentrations of ethanol and subsequently freeze-dried for 48 h. The cells were then affixed to the SEM sample stage using double-sided carbon conductive tape, coated with gold for 90 s at 12 mA, and observed using SEM (Tescan Company, Brno, Czech Republic).

#### 2.4.3. Observation Using a Transmission Electron Microscopy

Transmission electron microscopy (TEM) samples were prepared according to established protocols [25], with some modifications. Cells cultured on PDB medium for 3 days were initially treated similarly to those intended for SEM. The cleaned cells were fixed with 1% (*w*/*v*) osmium tetroxide (OsO_4_) at 4 °C for 2 h. Subsequently, the cells were dehydrated with increasing concentrations of ethanol. After dehydration, the cells were infiltrated with embedding medium using varying ratios of acetone. The embedded cells were left overnight at 37 °C, polymerized at 60 °C for 48 h, and sectioned into 1.5 μm semi-thin slices. These slices were stained with toluidine blue and examined under a light microscope. Subsequently, the sections were further cut into 60–80 nm ultra-thin slices and collected on copper mesh grids. The ultra-thin slices were stained in the dark with saturated 2% uranyl acetate solution in ethanol for 8 min. After washing, they were stained with a 2.6% lead citrate solution for 8 min in a CO_2_-free environment, washed again, and dried overnight at room temperature prior to observation with a TEM (HT7800/HT7700, Hitachi, Tokyo, Japan).

### 2.5. Measurement of Biomass, MPs Content, and CIT Content

The biomass (g/L) was determined as dry weight (DW) per liter of fermentation broth. The production of MPs was quantified using ultraviolet spectrophotometer (UV2600, Shimadzu, Kyoto, Japan) and reported as absorbance per gram (U/g) of dry mycelia. CIT content was assessed using high-performance liquid chromatography (HPLC; Waters, Milford, MA, USA). The analytical method is elaborated in detail by Xiong et al. [26], with minor modifications. Following the fermentation process, the fermentation broth was homogenized at 10,000 rpm for 1 min using a high-speed homogenizer. A suitable volume of homogenate was then treated with ethanol to extract MPs. CIT was extracted from the homogenate using a toluene/ethyl acetate/formic acid (7:3:1, v:v:v) mixture. The DW was quantified after the homogenate was dried to a constant weight at 60 °C.

### 2.6. Measurement of Iron Content

The iron content was determined using the phenanthroline iron complex colorimetric method. Following fermentation, the supernatant and mycelia were separated to analyze intracellular and extracellular iron contents. An equal volume of ultrapure water was added to the mycelium, which was subsequently homogenized using a high-speed homogenizer. Ammonium ferrous sulfate served as the standard solution for constructing a standard curve. A 500 μL sample of either the mycelial homogenate or fermentation supernatant was collected, followed by the sequential addition of 100 μL of 10 g/L hydroxylamine hydrochloride and 200 μL of phenanthroline (0.1 g/mL) solution. The mixture was thoroughly shaken at 37 °C, and after allowing the reaction to proceed for 30 min, it was centrifuged at 8000 rpm for 10 min. Subsequently, 500 μL of the supernatant was combined with 100 μL of concentrated hydrochloric acid and allowed to stand for 1 min to eliminate pigment interference. Absorbance was measured at 510 nm using a spectrophotometer, and the intracellular and extracellular iron content was calculated using the standard curve equation (*y* = 0.1613*x* + 0.1373, *R*^2^ = 0.9999).

### 2.7. RT-qPCR

The RELs of the corresponding gene were analyzed using ChamQ Universal SYBR qPCR Master Mix kit (Vazyme Biotech Co., Ltd., Nanjing, China) and the SLAN fluorescence detection system (SLAN, Shanghai, China), with GAPDH employed as the reference gene (primers are listed in Supplementary Table S3).

### 2.8. Statistical Analyses

Statistical figures were generated using Origin (Version 8.5), and statistical analysis was performed using one-way ANOVA in SPSS Statistics (Version 27.0). Data are presented as mean ± standard deviation based on five repeated measurements. Significant differences were determined at *p* < 0.05.

## 3. Results

### 3.1. The REL of the IscA1 Gene in RNAi-IscA1 and IscA1^+^ Strain

Following the establishment of RNAi-*IscA1* and *IscA1*^+^ strains, we analyzed the REL of the *IscA1* gene in these strains on the 4th day of fermentation (Supplementary Figure S3). In the *IscA1*^+^ strain, the REL was 124.1% of that in the WT strain, demonstrating a statistically significant upregulation (*p* < 0.05). Conversely, in the RNAi-*IscA1* strain, the REL was only 51.3% of that in the WT strain, indicating a substantial downregulation. These findings confirm significant differences in *IscA1* gene RELs among the three strains and validate the construction of the RNAi-*IscA1* and *IscA1*^+^ strains.

### 3.2. Colony Morphology, Microscopic Characteristics, and Cellular Structure of IscA1^+^ and RNAi-IscA1 Strains

#### 3.2.1. Colony Morphology of *IscA1*^+^ and RNAi-*IscA1* Strains on PDA Medium

**Figure 1:**
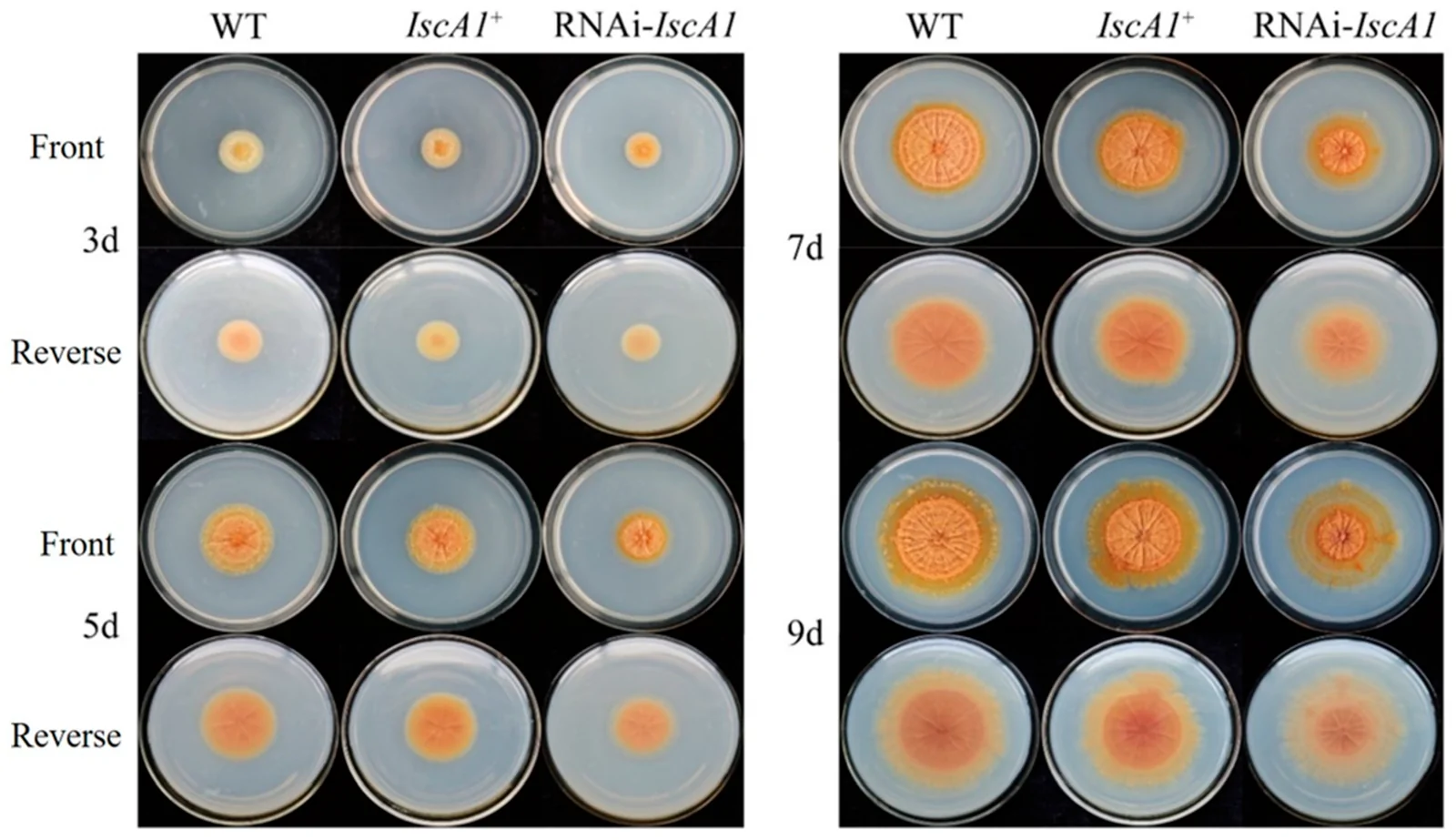
Colony morphology of *IscA1*^+^ and RNAi-*IscA1* strains on PDA medium. WT, wild type; *IscA1*^+^, *IscA1* overexpression strain; RNAi-*IscA1*, RNA interference strain. The note is identical in the following figure.

Daily observations were conducted to assess the colony morphology of various strains on PDA medium (Figure 1). After 7 days of solid culture, the RNAi-*IscA1* strain exhibited a distinct pattern of layered growth, characterized by restricted radial expansion and uneven distribution. This phenomenon was less pronounced in the WT and *IscA1*^+^ strains. Following 9 days of culture on PDA medium, the aerial hyphae of both the *IscA1*^+^ and RNAi-*IscA1* strains were darker than those of the WT. However, the RNAi-*IscA1* strain produced relatively fewer aerial hyphae compared to the WT.

*IscA1*^+^ strain exhibits normal growth on PDA medium, and throughout the entire cultivation period, there was no significant difference in colony size between the *IscA1*^+^ and WT strain (Figure 1; Table 1). Although the RNAi-*IscA1* strain can grow on PDA media, its colony diameter is significantly smaller than that of the WT starting on the 5th day of culture (Table 1). These results suggest that upregulating the expression of the *IscA1* gene does not influence the normal growth of *M. purpureus* on PDA medium. However, downregulating the expression of the *IscA1* gene adversely affects this fundamental life activity.

#### 3.2.2. Microscopic Characteristics of *IscA1*^+^ and RNAi-*IscA1* Strains on PDA/CYA Medium

**Figure 2:**
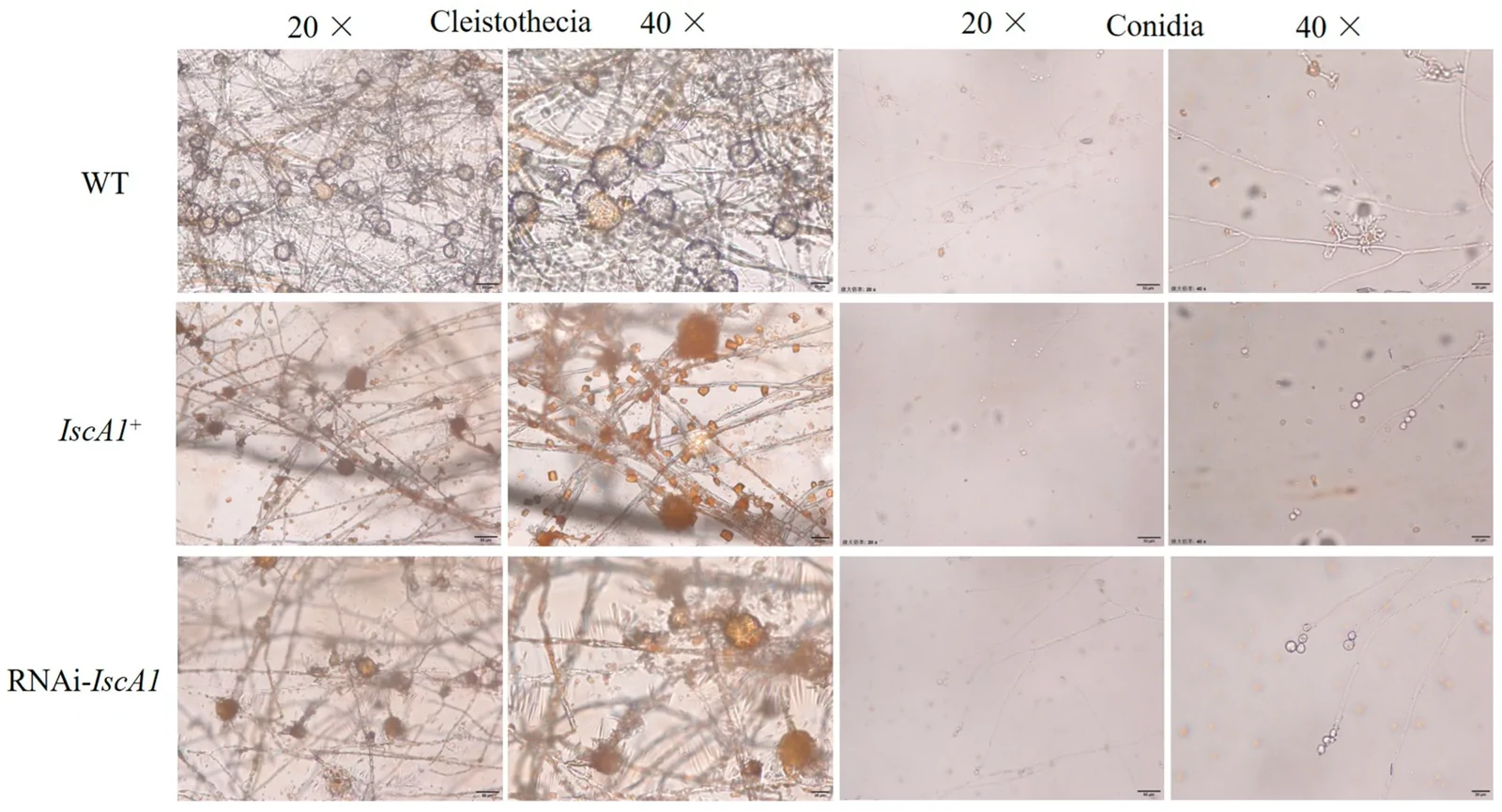
Microscopic characteristics of *IscA1*^+^ and RNAi-*IscA1* strains cultivated on PDA medium for 8 days.

On PDA medium, the WT, *IscA1*^+^, and RNAi-*IscA1* strains can all produce cleistothecia and conidia; however, the number of cleistothecia produced by the *IscA1*^+^ and RNAi-*IscA1* strains is obvious lower than that of the WT. There is no distinct difference in the number of conidia produced by the *IscA1*^+^ and RNAi-*IscA1* strains compared to the WT (Figure 2). These findings indicate that the sexual reproduction of the *IscA1*^+^ and RNAi-*IscA1* strains was affected, whereas asexual reproduction was not influenced.

**Figure 3:**
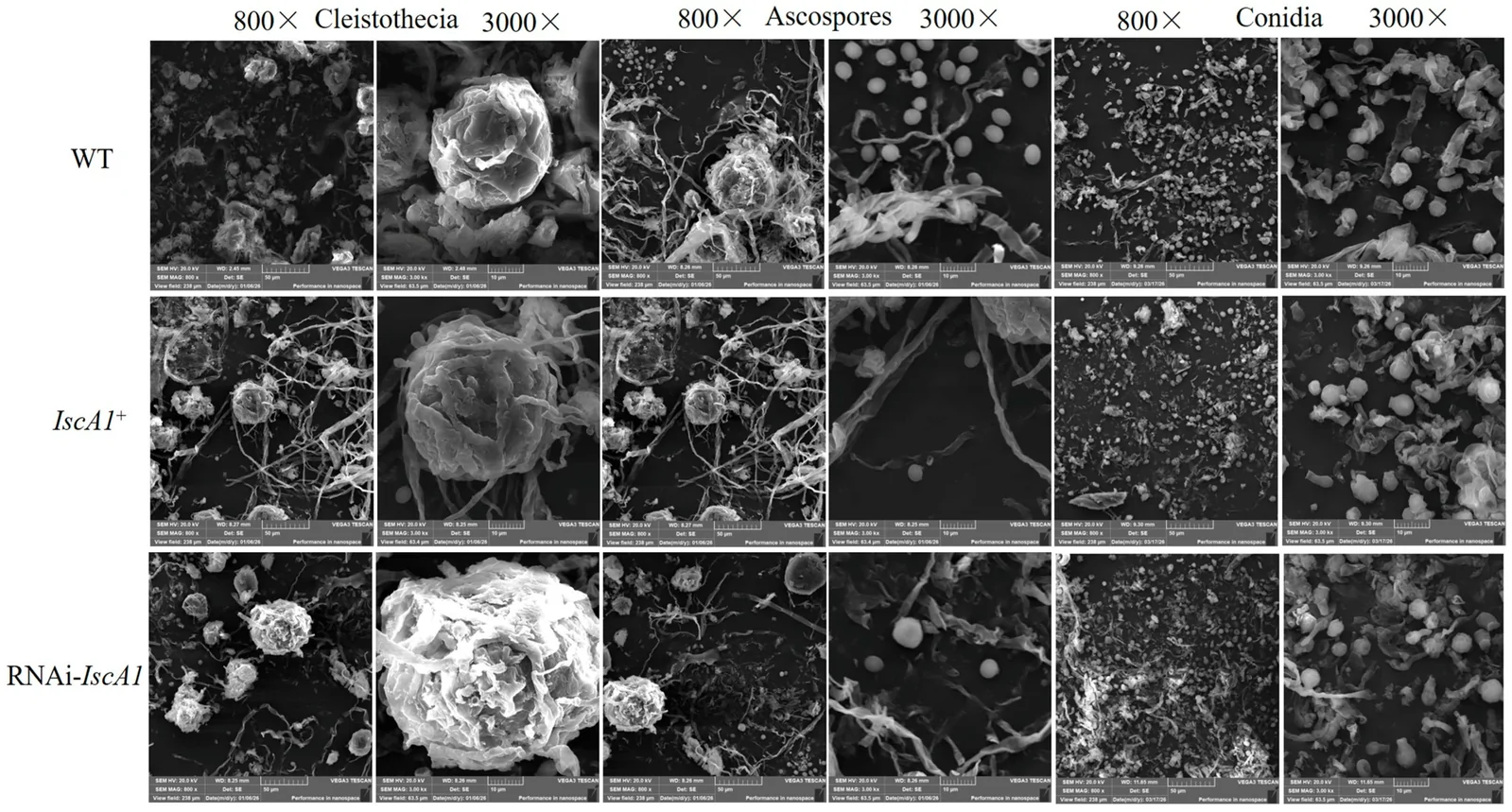
Cleistothecia, ascospores, and conidia of *IscA1*^+^ and RNAi-*IscA1* strains using SEM.

SEM was employed to examine various strains cultivated on PDA and CYA for 8 days to identify differences in the microstructures of cleistothecia, ascospores, and conidia (Figure 3). At a magnification of 800×, the number of cleistothecia in the *IscA1*^+^ strain was obvious lower than that in the WT, with RNAi-*IscA1* exhibiting a similar pattern. When assessing the surface morphology of cleistothecia at 3000× magnification, the appearances across different strains were relatively consistent, all were enveloped in mycelium and exhibited closed spherical shapes with uneven surfaces.

Observations of the ascospores at a magnification of 800× revealed no distinct differences in abundance among the three strains. At a magnification of 3000×, all ascospores were uniformly oval-shaped, exhibiting smooth surfaces devoid of wrinkles, depressions, or other abnormalities, and showed no notable variations. The mycelium from each strain was smooth and robust, with no abnormal phenotypic characteristics observed.

At 800× magnification, no distinct difference was observed in the number of conidia among the three strains across the entire field of view. Further examination of conidial morphology at 3000× magnification revealed that most conidia from diverse strains exhibited an inverted pear shape, while a minority appeared round. However, conidia from different strains displayed slight shrinkage, likely resulting from inadequate gradient dehydration and freeze-drying techniques. This finding suggests that there are no overall differences in the morphology of conidia among the three strains.

#### 3.2.3. Cell Morphology and Organelle Structure of *IscA1*^+^ and RNAi-*IscA1* Strains

**Figure 4:**
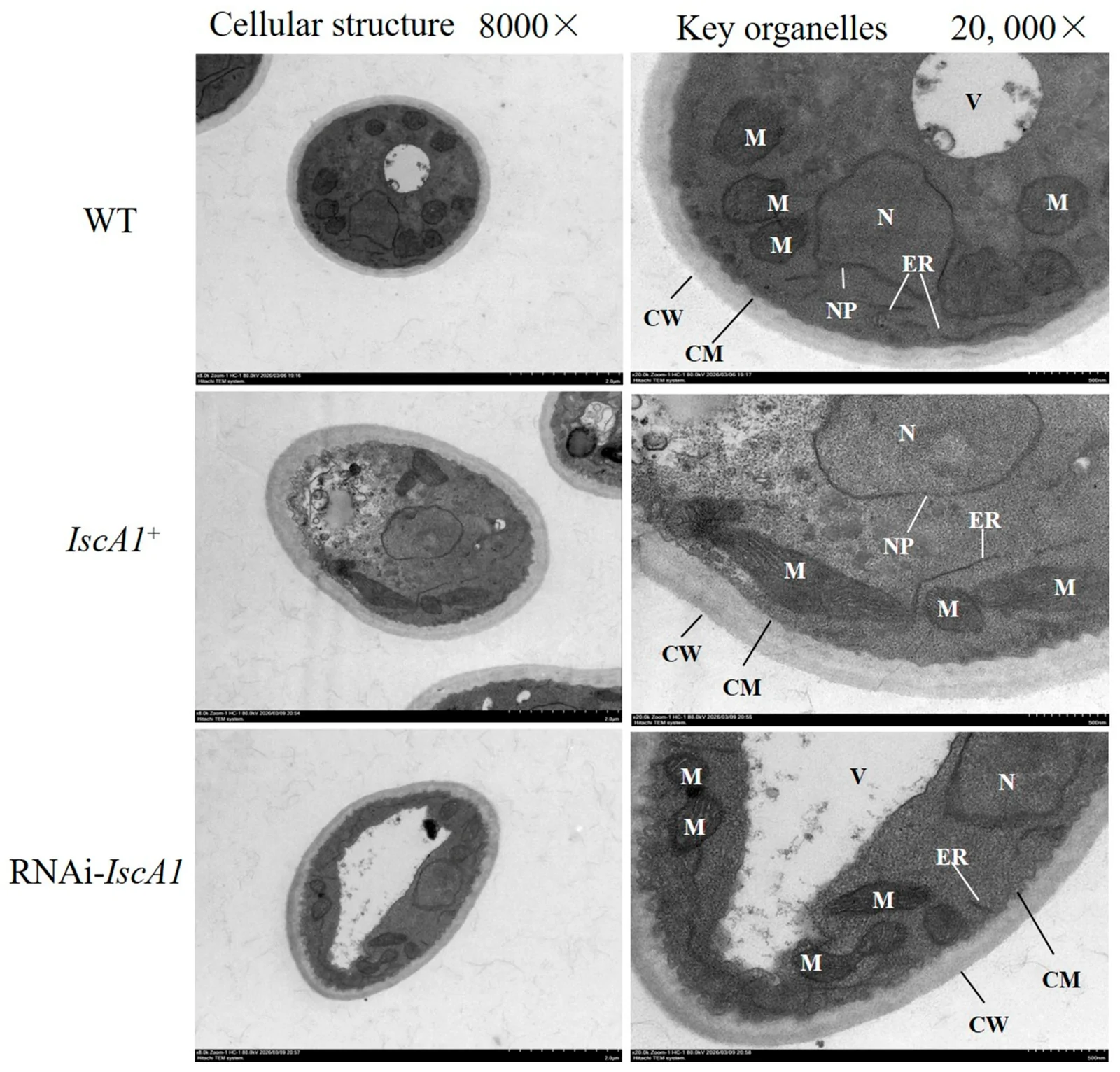
Cellular structural and morphological characteristics of *IscA1*^+^ and RNAi-*IscA1* strains using TEM. CW, cell wall; CM, cell membrane; V, vacuole; M, mitochondrion; N, nucleus; NP, nuclear pore; ER, endoplasmic reticulum.

TEM was employed to investigate the cell morphology and organelle structure of the three strains. At a magnification of 8000×, the overall cellular architectures of all three strains appeared intact, with no observable damage to critical cellular components, including the cell wall, cell membrane, and nucleus (Figure 4).

Significant vacuole structures were identified in the center of the WT and RNAi-*IscA1* cells; however, no prominent central vacuoles or small vacuole structures were detected in the *IscA1*^+^ strain. This discrepancy may be attributed to the physiological differentiation occurring during liquid fermentation, where cells at the periphery of the mycelial aggregates are more metabolically active, resulting in smaller vacuoles that are obscured by the limitations of planar sectioning [27]. In contrast, the cells located within the mycelial aggregates exhibited signs of aging, leading to the fusion and enlargement of vacuoles, which became prominent organelle structures. Observations at 20,000× magnification of primary cellular structures and mitochondria confirmed that the cell wall, cell membrane, and nucleus of the three strains remained intact, and complete mitochondrial structures were distinctly visible, including both intact inner and outer membrane structures and parallel-arranged cristae.

### 3.3. The Biomass, MPs and CIT Production of IscA1^+^ and RNAi-IscA1 Strains

**Figure 5:**
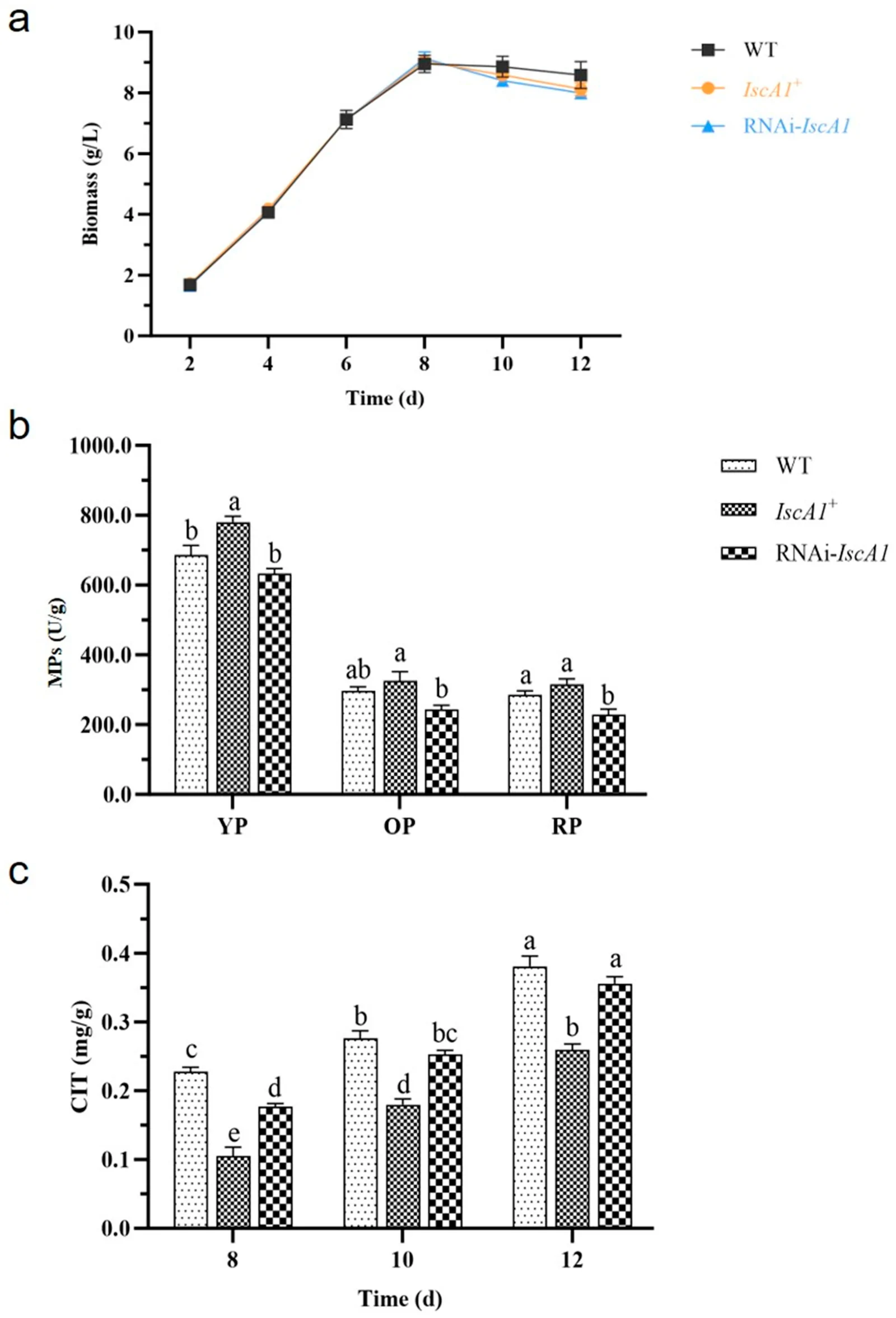
The biomass, MPs and CIT production of *IscA1*^+^ and RNAi-*IscA1* strains. (**a**) Biomass; (**b**) MPs production on the 6th day of liquid fermentation. YP, yellow pigments production; OP, orange pigments production; RP, red pigments production. (**c**) CIT production on the 8th, 10th, and 12th days of liquid fermentation. Different lowercase letters above the columns indicate significant differences (*p* < 0.05).

The biomass, the production of MPs on the 6th day and the CIT production on the 8th, 10th, and 12th days were measured. As fermentation progressed, the biomass of the WT, *IscA1*^+^ and RNAi-*IscA1* strains increased gradually. In the early phase of fermentation, biomass exhibited rapid growth, peaking on the 8th day before experiencing a slight decline. Throughout the fermentation process, no significant differences in biomass were observed among the three strains (Figure 5a).

On the 6th day of fermentation, the YP production of the *IscA1*^+^ strain increased by 18.2% compared to the WT, while no significant differences were noted in the production of OP and RP. Additionally, the RP production in the RNAi-*IscA1* strain decreased by 22.8% compared to the WT, although no significant differences in YP and OP production were observed between the WT and RNAi-*IscA1* (Figure 5b). The CIT production of the *IscA1*^+^ strain was significantly lower than that of the WT, with reductions of 53.2%, 36.8% and 38.5% on the 8th, 10th, and 12th days, respectively. Similarly, on the 8th day, the CIT production of the RNAi-*IscA1* was significantly lower than that of the WT, exhibiting a decrease of 20.5%. However, no significant differences were observed on the 10th and 12th days (Figure 5c). On the 8th day of fermentation, both the *IscA1*^+^ and RNAi-*IscA1* strains showed significantly lower CIT production compared to the WT, indicating that both overexpression and underexpression of the *IscA1* gene effectively inhibit CIT synthesis at this stage of fermentation.

### 3.4. Effect of LF-MF on the Biomass, MPs and CIT Production of IscA1^+^ and RNAi-IscA1 Strains

**Figure 6:**
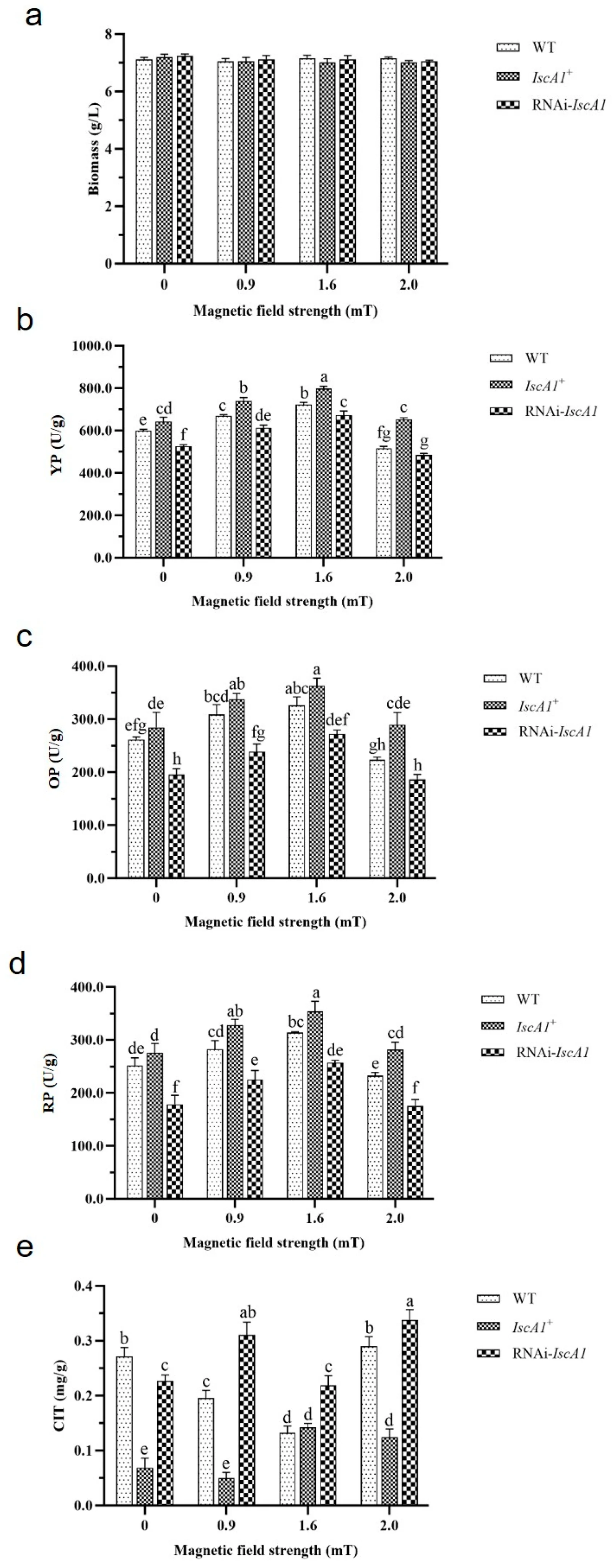
Effect of LF-MF exposure on the biomass, MPs and CIT production of *IscA1*^+^ and RNAi-*IscA1* strains. (**a**) Biomass; (**b**) YP production; (**c**) OP production; (**d**) RP production; (**e**) CIT production. Different lowercase letters above the columns indicate significant differences (*p* < 0.05).

Exposure to varying intensities of LF-MF did not significantly influence the biomass of *IscA1*^+^, and RNAi-*IscA1* strains during liquid fermentation (Figure 6a). However, it had a substantial impact on MPs production and CIT production. LF-MF intensities of 0.9 mT and 1.6 mT enhanced MPs production across various strains, while a 2.0 mT LF-MF inhibited MP production (Figure 6), with the 1.6 mT LF-MF exhibiting the most pronounced promoting effect on MP production. The influences of LF-MF on YP, OP and RP production were consistent among different strains (Figure 6b–d). Specifically, the 1.6 mT LF-MF resulted in increases in YP production by 21.1%, 18.5% and 31.9%, OP production by 25.1%, 19.0%, and 31.8%, and RP production by 25.4%, 24.7%, and 36.7% for the WT, *IscA1*^+^, and RNAi-*IscA1* strains.

The effects of exposure to varying intensities of LF-MF on the CIT production of *IscA1*^+^ and RNAi-*IscA1* strains are differing (Figure 6e). Exposure to LF-MF at intensities of 0.9 mT and 1.6 mT significantly reduces the CIT production of the WT by 28.8% and 51.1%, respectively. Conversely, exposure to LF-MF at intensities of 1.6 mT and 2.0 mT significantly enhances the CIT production of *IscA1*^+^ strain, with increases of 102.4% and 76.0%, respectively. Additionally, exposure to LF-MF at 0.9 mT and 2.0 mT significantly boosts the CIT production of RNAi-*IscA1*, resulting in increases of 34.5% and 45.2%, respectively. Notably, exposure to 1.6 mT LF-MF reduces the CIT production of WT while increasing production in the *IscA1*^+^ strain.

### 3.5. Effect of LF-MF on Iron Content in IscA1^+^ and RNAi-IscA1 Strains

**Figure 7:**
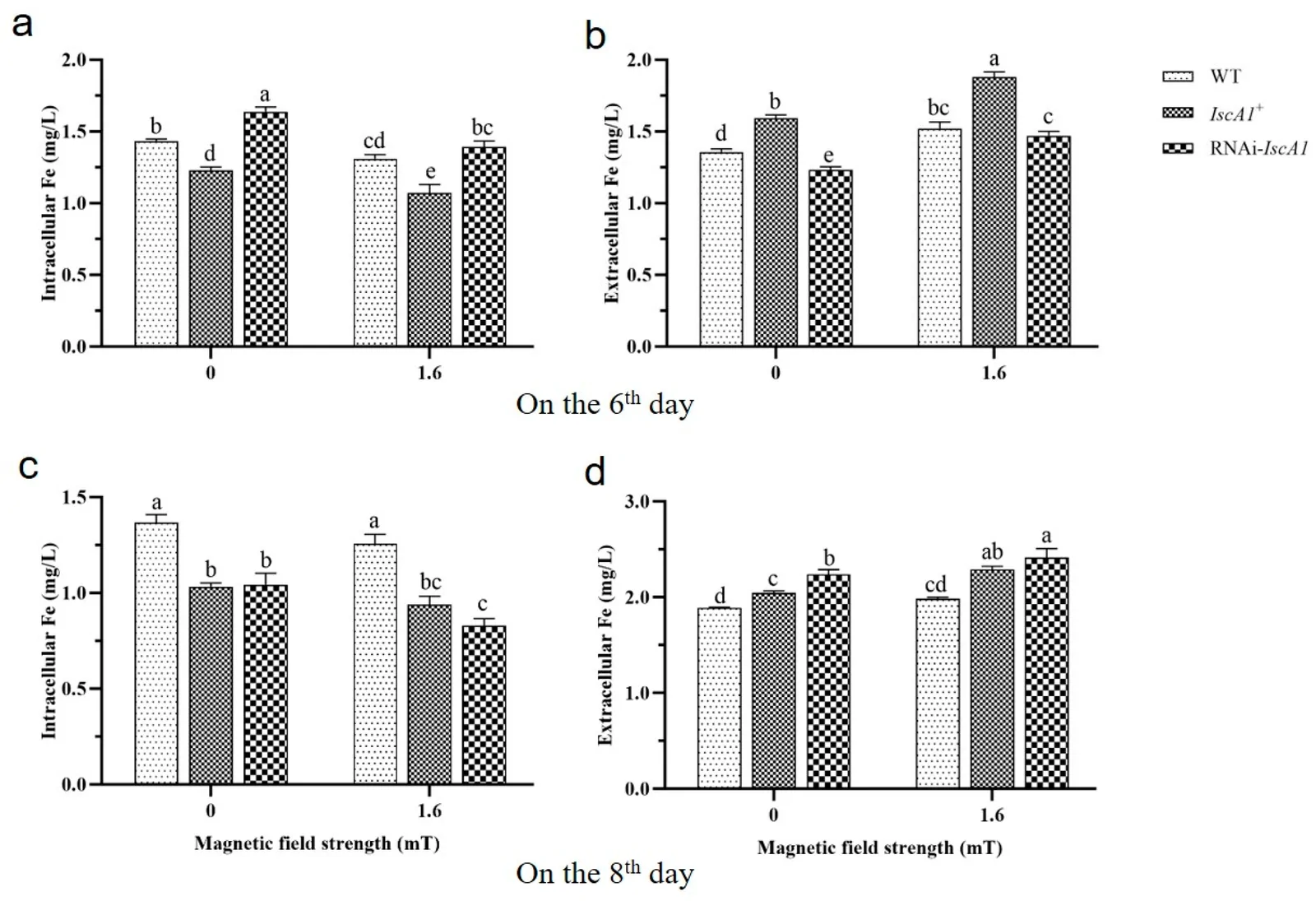
Effect of LF-MF exposure on the intracellular and extracellular iron content in *IscA1*^+^ and RNAi-*IscA1* strains. (**a**,**c**) Intracellular iron content; (**b**,**d**) extracellular iron content. Different lowercase letters above the columns indicate significant differences (*p* < 0.05).

The *IscA1*^+^ and RNAi-*IscA1* strains exhibit distinct differences in intracellular and extracellular iron content throughout the fermentation stages. On the 6th day of fermentation, the intracellular iron content in the *IscA1*^+^ strain is significantly lower than that in the WT, while the intracellular iron content in the RNAi-*IscA1* exhibits significantly higher intracellular iron content compared to the WT (Figure 7a). Conversely, the extracellular iron content in the *IscA1*^+^ is significantly elevated compared to the WT, whereas the extracellular iron content in the RNAi-*IscA1* is significantly reduced relative to the WT (Figure 7b). Following exposure to 1.6 mT LF-MF, the intracellular iron content of all three strains decreased significantly compared to the untreated group; while the extracellular iron content shows a significant increase. However, no significant differences in either intracellular or extracellular iron content are observed between the RNAi-*IscA1* and the WT strains after exposure to LF-MF. The intracellular iron content in the *IscA1*^+^ strain remains significantly lower than that of the WT, whereas the extracellular iron content remains significantly higher. On 8th day of fermentation, both the *IscA1*^+^ and RNAi-*IscA1* strains exhibit significantly lower intracellular iron content than the WT (Figure 7c), while their extracellular iron content is significantly higher (Figure 7d). After exposure to 1.6 mT LF-MF, the intracellular iron content in the RNAi-*IscA1* strain significantly decreases, while the extracellular iron content shows a significant increase. Notably, exposure to a 1.6 mT LF-MF had no significant effect on the intracellular iron content in the WT and *IscA1*^+^ strain.

### 3.6. Effect of LF-MF on the Expression of Related Genes in IscA1^+^ and RNAi-IscA1 Strains

**Figure 8:**
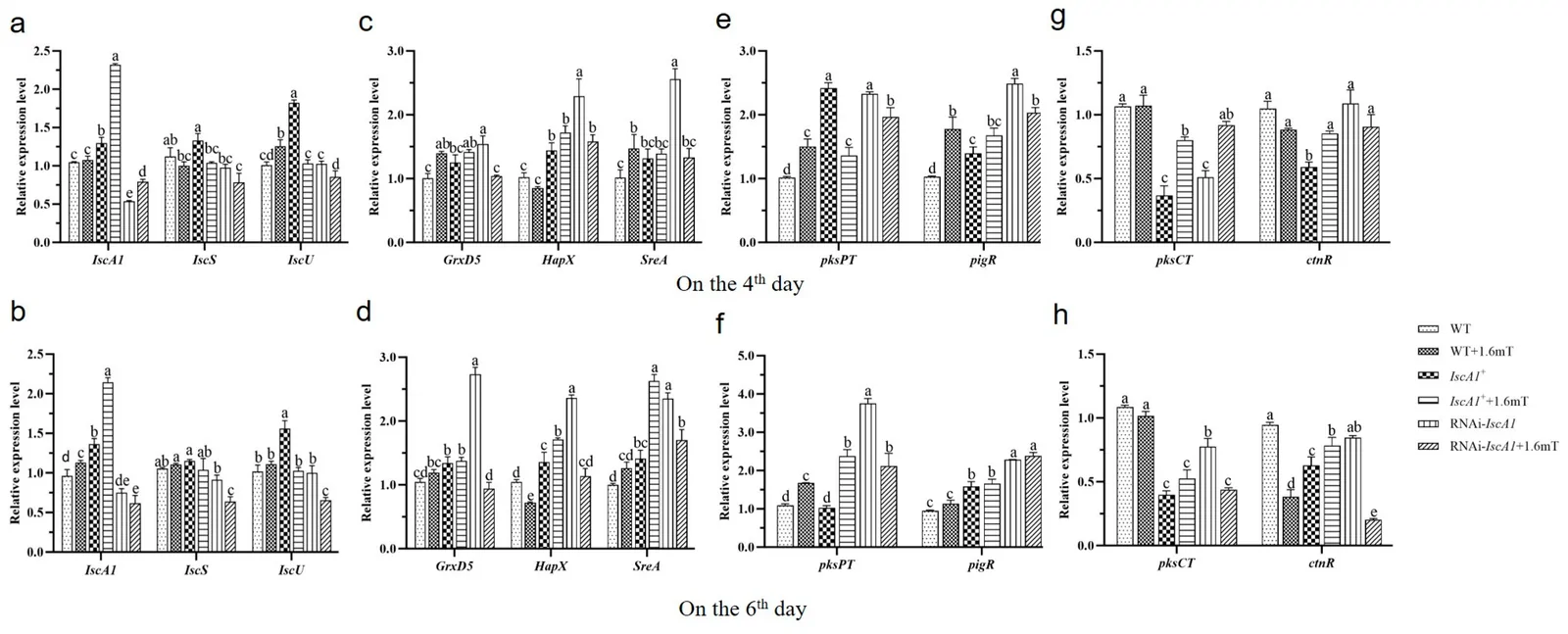
The REL of genes associated with iron–sulfur cluster assembly system, iron metabolism, MPs and CIT synthesis in *IscA1*^+^ and RNAi-*IscA1* strains on the 4th and 6th day of fermentation. (**a**,**b**) The REL of genes associated with iron–sulfur cluster assembly system; (**c**,**d**) the REL of genes associated with iron homeostasis; (**e**,**f**) the REL of genes associated with MPs synthesis; (**g**,**h**) the REL of genes associated with CIT synthesis. Different lowercase letters above the columns of the same gene indicate significant differences (*p* < 0.05).

Exposure to LF-MF significantly influences the RELs of key genes involved in the iron–sulfur cluster assembly system in *IscA1*^+^ and RNAi-*IscA1* strains. On the 4th day of fermentation, LF-MF exposure resulted in the upregulation of the *IscA1* gene expression in both *IscA1*^+^ and RNAi-*IscA1* strains, as well as the *IscU* gene expression in WT. In contrast, the *IscS* gene expression was downregulated in *IscA1*^+^, while *IscU* gene was also downregulated in the RNAi-*IscA1* strain (Figure 8a). On the 6th day of fermentation, LF-MF exposure again led to the upregulation of the *IscA* gene expression in *IscA1*^+^ strain, while simultaneously downregulating *IscS* gene in RNAi-*IscA1* and *IscU* gene in both *IscA1*^+^ and RNAi-*IscA1* strains (Figure 8b). Furthermore, the overexpression and underexpression of the *IscA1* gene exert differential effects on the expression of key genes in the iron–sulfur cluster assembly system. Specifically, on the 4th day of fermentation, compared to WT, the *IscA1*^+^ strain exhibited upregulation of *IscA1* gene expression, whereas the RNAi-*IscA1* strain displayed downregulation. However, no similar changes were observed in the *IscS* gene expression. Conversely, *IscU* gene upregulation was observed exclusively in *IscA1*^+^ strain. On the 6th day of fermentation, significant upregulation of *IscA1* and *IscU* gene expression was noted only in *IscA1*^+^ strain compared to the WT.

Exposure to LF-MF resulted in significant variation in the RELs of iron homeostasis-related genes in *IscA1*^+^ and RNAi-*IscA1* strains. On the 4th day of fermentation, LF-MF exposure resulted in an upregulation of *GrxD5* and *SreA* gene expression in the WT, while it downregulated the expression of *GrxD5*, *HapX*, and *SreA* genes in the RNAi-*IscA1* strain (Figure 8c). On the 6th day of fermentation, LF-MF exposure similarly upregulated the expression of the *HapX* and *SreA* genes in the *IscA1*^+^ strain. In addition, there was a simultaneous downregulation of HapX gene expression in the WT strain, apart from the genes that were downregulated on the 4th day of fermentation (Figure 8d). Furthermore, notable differences were observed in the expression of iron homeostasis-related genes in *IscA1*^+^ and RNAi-*IscA1* strains at different fermentation times. Specifically, on the 4th day of fermentation, the expression of these three genes was upregulated in the RNAi-*IscA1* strain when compared to the WT, while *HapX* was upregulated in the *IscA1*^+^ strain. Conversely, on the 6th day of fermentation, the RELs of these three genes were all upregulated in the *IscA1*^+^ and RNAi-*IscA1* compared to the WT.

Exposure to LF-MF significantly affects the RELs of the regulatory genes involved in MPs synthesis in *IscA1*^+^ and RNAi-*IscA1* strains. On the 4th day of fermentation, LF-MF exposure resulted in an upregulation of *pksPT* and *pigR* gene expression in the WT. In contrast, *pksPT* gene expression was downregulated in the *IscA1*^+^, as well as in both *pksPT* and *pigR* gene in the RNAi-*IscA1* strain (Figure 8e). On the 6th day of fermentation, changes were observed only in the RELs of the *pksPT* gene. LF-MF exposure caused an increase in the RELs of *pksPT* in both the WT and *IscA1*^+^ strains, while it resulted in downregulation in the RNAi-*IscA1* strain (Figure 8f). Furthermore, notable differences in the expression of genes involved in MPs synthesis were identified between *IscA1*^+^ and RNAi-*IscA1* strains at different fermentation times. On the 4th day of fermentation, RELs of *pksPT* increased in both *IscA1*^+^ and RNAi-*IscA1* strains compared to the WT, while the expression of *pigR* was also upregulated in the RNAi-*IscA1* strain. Conversely, on the 6th day of fermentation, the RELs of *pksPT* in RNAi-*IscA1* strain increased relative to the WT, as did the RELs of *pigR* in both *IscA1*^+^ and RNAi-*IscA1* strains.

Exposure to LF-MF influenced the RELs of genes regulating CIT synthesis in *IscA1*^+^ and RNAi-*IscA1* strains. On the 4th day of fermentation, LF-MF exposure upregulated the expression of the *pksCT* and *ctnR* genes in the *IscA1*^+^ strain, as well as the *pksCT* gene in the RNAi-*IscA1* strain (Figure 8g). On the 6th day of fermentation, LF-MF exposure continued to upregulate the expression of the *ctnR* gene in the *IscA1*^+^ strain, while simultaneously downregulating the *ctnR* gene in the WT and the expression of both *pksCT* and *ctnR* in the RNAi-*IscA1* strain (Figure 8h). Additionally, differences in the expression of genes regulating CIT synthesis were observed between *IscA1*^+^ and RNAi-*IscA1* strains. Specifically, on the 4th and 6th days of fermentation, the expression levels of the *pksCT* and *ctnR* genes in the *IscA1*^+^ strain, as well as the *pksCT* gene in the RNAi-*IscA1* strain, were downregulated compared to the WT.

## 4. Discussion

The study reveals that there is no significant difference in colony size between the *IscA1*^+^ and WT strains when cultivated on PDA. This finding suggests that a moderate increase in *IscA1* gene expression can maintain a balance in the intracellular supply of iron–sulfur clusters without interfering with mycelial proliferation. Although the RNAi-*IscA1* strain can grow on PDA medium, it exhibits a phenotype characterized by inhibited radial growth and uneven development as solid cultures progress to later developmental stages. This effect may result from reduced expression of the *IscA1* gene, which impacts iron–sulfur cluster synthesis and leads to abnormal accumulation of free iron within cells, thereby inducing oxidative stress that affects the physiological functions of fungal mycelial and colony expansion [28,29]. The cell wall, cell membrane, nucleus, and mitochondria of both *IscA1*^+^ and RNAi-*IscA1* strains remain intact, and the structure of the mitochondrial inner membrane cristae is undamaged, demonstrating that perturbations in *IscA1* do not compromise the cytoskeleton or respiratory core organelles [30].

In terms of reproductive development, both the overexpression and underexpression of *IscA1* have been shown to selectively inhibit sexual reproduction without affecting asexual conidium formation. The spore morphology of *IscA1*^+^ and RNAi-*IscA1* does not exhibit abnormalities. However, the number of cleistothecia utilized for sexual reproduction is significantly reduced. Asexual reproduction in *M. purpureus* relies on fundamental carbon metabolism and cell wall synthesis pathways [31], where many key enzymes are not dependent on iron–sulfur cluster cofactors, thereby rendering them unaffected by disturbances in *IscA1* expression. Conversely, the sexual developmental pathway associated with cleistothecium formation is regulated by intracellular iron signals [32,33,34]. The disruption in iron homeostasis mediated by *IscA1* can downregulate genes related to sexual development through the iron-responsive transcription factors HapX and SreA [32], selectively inhibiting cleistothecium differentiation without affecting spore morphology. This underscores the selective regulatory role of *IscA1* in fungal development.

Throughout the fermentation period, no significant differences in biomass were observed between WT, *IscA1*^+^, and RNAi-*IscA1* strains in liquid fermentation. This finding indicates that *IscA1* specifically targets and regulates iron-dependent secondary metabolism without contributing to fundamental nutritional processes such as carbon source utilization and cell proliferation, thereby enabling the decoupled regulation of nutritional growth and secondary metabolic pathways. However, the response of MPs production to changes in *IscA1* gene expression exhibited component-specific characteristics. On the 6th day of fermentation, YP production in *IscA1*^+^ strain increased, whereas RP production in RNAi-*IscA1* strain decreased. IscA1 functions as a mitochondrial iron–sulfur cluster carrier protein, facilitating the transfer of iron–sulfur clusters from scaffold proteins to target proteins, which is critical for maintaining energy metabolism, iron homeostasis, and the activity of key enzymes. Variations in *IscA1* gene expression levels may induce an imbalance in intracellular iron homeostasis and the redox environment [35,36]. The activity of the reductase MppE is sensitive to the redox conditions, and its enhanced activity has been shown to specifically dominate the directed synthesis of YP while inhibiting the metabolic flow toward the RP synthesis branch [36,37]. Furthermore, real-time quantitative results revealed that on the 4th and 6th day of fermentation, the expression levels of the pigment synthesis gene *pksPT* and regulatory gene *pigR* in *IscA1*^+^ and RNAi-*IscA1* strains were generally higher than those in the WT, suggesting that the regulation of MPs synthesis-related genes by *IscA1* expression may involve a dose-dependent effect or feedback regulatory mechanisms.

The synthesis of CIT is significantly influenced by the expression of *IscA1*, with notable time-dependent differences among strains. The production of CIT in *IscA1*^+^ strain was markedly lower than that of the WT. In contrast, the suppression of CIT synthesis in the RNAi-*IscA1* strain was observed only on the 8th day of fermentation, with no significant difference compared to the WT in the subsequent stages. At the molecular level, the transcription levels of the key gene *pksCT* and the regulatory gene *ctnR* involved in CIT synthesis in *IscA1*^+^ and RNAi-*IscA1* strains remained consistently lower than those in the WT, indicating impeded CIT synthesis. From the perspective of iron distribution, *IscA1*^+^ strains utilize a substantial amount of intracellular free iron for iron–sulfur cluster assembly; this iron deficiency signal prompts the cell to prioritize energy supply, consequently inhibiting the synthesis of secondary metabolites [38,39]. In the early phase, the intracellular iron overload in the RNAi-*IscA1* strain triggers a metabolic shift from primary to secondary metabolism [40], while also facilitating compensatory regulation of iron homeostasis through the re-localization of intracellular iron and inhibition of iron uptake [41,42], thereby moderating iron distribution.

The analysis of intracellular and extracellular iron content confirms that *IscA1* is a pivotal gene regulating iron transport and distribution in *M. purpureus*. Variation in *IscA1* expression levels results in two distinct patterns of iron distribution. On the 6th day of fermentation, intracellular iron content in *IscA1*^+^ strain significantly decline, while extracellular iron accumulates. This phenomenon may be linked to cellular responses to iron availability. Research indicates that fungi dynamically modulate their iron uptake intensity during iron homeostasis by sensing the status of iron–sulfur clusters [43,44,45]. In the *IscA1*^+^ strain, cells continuously transport free iron for iron–sulfur cluster assembly and utilize monothiol glutaredoxin to regulate the expression of iron uptake genes, thereby preventing iron overload [43,46] and reducing intracellular iron content. In the RNAi-*IscA1* strain, impaired iron–sulfur synthesis causes the cells to misinterpret the state as “iron deficiency,” resulting in excessive iron uptake and abnormal intracellular iron accumulation [44]. When intracellular iron levels become excessive, the cell activates compensatory mechanisms to regulate iron homeostasis [47,48]. On the 8th day of fermentation, intracellular iron content in the RNAi-*IscA1* strain decrease, aligning its iron distribution with that of *IscA1*^+^. Thus, intracellular iron content serves as an intermediate signaling molecule that synchronously regulates the transcription of four major gene categories: iron–sulfur assembly, iron homeostasis, MPs synthesis, and CIT synthesis, positioning it as a key mediator between *IscA1* gene expression and macro- and metabolic phenotypes.

Exposure to various intensities of LF-MF did not result in significant changes in the biomass of the three strains, suggesting that the LF-MF acts merely as an external physical signal to regulate iron homeostasis and secondary metabolism, without interfering with the fundamental proliferation of *M. purpureus*. The LF-MF exerts a gradient effect on the production of MPs; an intensity of 1.6 mT demonstrated the most pronounced enhancement of YP, OP, and RP synthesis among the three strains. On the 6th day of fermentation, following exposure to the 1.6 mT LF-MF, intracellular iron content significantly decreased. Consequently, the LF-MF may influence the absorption of iron by *M. purpureus* cells by altering the membrane potential or interfering with the conformation of membrane transport proteins [49]. This alteration may lead to a reduction in intracellular free iron levels, a weakening of the cells’ antioxidant capacity [50], and an enhancement in MPs synthesis. These findings align with previous research indicating that the LF-MF regulates the synthesis of MPs through ROS [9].

The regulation of CIT by LF-MF exhibits significant strain dependence, linked to the expression of the *IscA1* gene. The same LF-MF intensity produces opposing effects on the WT, *IscA1*^+^, and RNAi-*IscA1* strains: exposure to 1.6 mT LF-MF significantly reduces CIT production in the WT strain while markedly increases CIT production in the *IscA1*^+^ strain. The *IscA1*^+^ strain demonstrates lower intracellular iron content, which decreases further after exposure to LF-MF, prompting the strain to compensatorily activate the CIT synthesis pathway. Real-time qPCR results indicate that the expression levels of *pksCT* and *ctnR* are significantly downregulated in the *IscA1*^+^ strain, where CIT synthesis is notably inhibited. The 1.6 mT LF-MF upregulates the expression of *pksCT* and *ctnR*, correlating with the substantial increase in CIT production in the *IscA1*^+^ strain following LF-MF exposure. This observation suggests that the expression level of *IscA1* alters the pathway’s response to LF-MF or intracellular iron content [51]. In contrast, the RNAi-*IscA1* strain exhibits higher intracellular iron content, which decreases after LF-MF exposure; however, the 1.6 mT LF-MF does not influence CIT synthesis in the RNAi-*IscA1* strain. Thus, the response of CIT synthesis to LF-MF exposure reveals a high level of complexity, necessitating further investigation into the mechanisms underlying this response, particularly regarding *IscA1* gene expression.

Based on the findings of this study, we hypothesize that IscA1 in *M. purpureus* is influenced by LF-MF signals that regulate the expression of genes associated with iron–sulfur cluster assembly and iron homeostasis. Consequently, this modulation alters intracellular iron distribution and homeostasis, thereby affecting the synthesis of MPs and CIT. Further investigation of the pathway targets and their underlying mechanisms is required to substantiate this hypothesis.

## 5. Conclusions

The overexpression and underexpression of the *IscA1* gene specifically regulate both nutritional growth and sexual reproduction in *M. purpureus*, without significantly affecting asexual reproduction. While these expressions did not alter the ultrastructure of spores and mycelia, they did lead to differences in the development of intracellular vacuoles. Furthermore, neither the overexpression nor the underexpression of the *IscA1* gene affected the biomass in WT, *IscA1*^+^, and RNAi-*IscA1* strains. The *IscA1*^+^ strain significantly increased YP production while markedly decreasing CIT production. Conversely, the RNAi-*IscA1* strain significantly reduced both the production of RP and CIT on the 8th day of fermentation. Exposure to LF-MF did not affect the biomass in liquid fermentation but significantly influenced the synthesis of MPs and CIT. Furthermore, exposure to a 1.6 mT LF-MF substantially enhanced the production of YP, OP, and RP, while reducing CIT production in the WT and significantly increasing CIT accumulation in the *IscA1*^+^ strain. On the 4th day of fermentation, the *IscA1*^+^ strain exhibited reduced intracellular iron content, whereas the RNAi-*IscA1* strain displayed the opposite trend. The 1.6 mT LF-MF exposure generally reduced intracellular iron content and altered the distribution of iron ions both intracellularly and extracellularly. These results suggest that the 1.6 mT LF-MF affects *IscA1* gene expression and regulates the expression of genes related to iron–sulfur cluster assembly, iron homeostasis, and the synthesis of MPs and CIT. The research findings provided a data reference for achieving the safe production of *Monascus* fermentation products through magnetic field-assisted fermentation methods.

## Figures and Tables

**Table 1:** Colony diameters of *IscA1*^+^ and RNAi-*IscA1* strains cultured on PDA medium.

Culture Time (d)	Colony Diameter (cm)						
	5	6	7	8	9	10	11
WT	3.6 ± 0.1 ^a^	4.4 ± 0.1 ^a^	5.3 ± 0.1 ^a^	5.9 ± 0.2 ^a^	6.6 ± 0.1 ^a^	6.9 ± 0.1 ^a^	7.5 ± 0.2 ^a^
*IscA1* ^+^	3.5 ± 0.1 ^a^	4.4 0.1 ^a^	5.3 ± 0.1 ^a^	6.0 ± 0.2 ^a^	6.8 ± 0.1 ^a^	7.1 ± 0.1 ^a^	7.6 ± 0.1 ^a^
RNAi-*IscA1*	3.1 ± 0.1 ^b^	3.9 ± 0.1 ^b^	4.6 ± 0.1 ^b^	5.1 ± 0.1 ^b^	6.0 ± 0.1 ^b^	6.4 ± 0.2 ^b^	7.0 ± 0.2 ^b^

Distinct superscript letters within the same column indicate statistically significant differences among the strains (*p* < 0.05).

## Data Availability

The original contributions presented in this study are included in the article/Supplementary Materials. Further inquiries can be directed to the corresponding author.

## Supplementary Materials

The following supporting information can be downloaded at https://www.mdpi.com/article/10.3390/jof12080585/s1, Figure S1: Construction of *IscA1* gene RNA interference vector; Figure S2: Construction of *IscA1* gene overexpression vector; Figure S3: The relative expression levels of *IscA1* gene in the *IscA1*^+^ and RNAi-*IscA1* strains; Table S1: Primers for constructing the *IscA1* gene RNA interference vector; Table S2: Primers for constructing the *IscA1* gene overexpression vector; Table S3: Primers of genes used for real-time quantitative PCR.

## Abbreviations

The following abbreviations are used in this manuscript:

MPs	*Monascus* pigments
*RNAi*-*IscA1*	*IscA1* gene RNA interference strains
*IscA1*^+^	*IscA1* gene overexpression strains
YP	yellow pigments
OP	orange pigment
RP	red pigment
CIT	citrinin
LF-MF	low-frequency magnetic field
ROS	reactive oxygen species
IscA	A-type iron–sulfur cluster protein
RELs	relative expression levels
PDA	potato dextrose agar
CYA	Czapek yeast extract agar
PDB	potato dextrose broth
AMT	*Agrobacterium*-mediated transformation
CDS	coding sequence
WT	wild-type
SEM	scanning electron microscopy
TEM	Transmission electron microscopy
DW	dry weight
