# Supplementary material for: Role of IscA1 in Low-Frequency Magnetic Field-Associated Changes in Monascus Pigments and Citrinin Levels in Monascus purpureus
Source: J Fungi (Basel). 2026 Aug 7;12(8):585. doi: 10.3390/jof12080585 (PMC13514923; doi:10.3390/jof12080585)
Supplement: Supplementary file 1 [file jof-12-00585-s001.zip › jof-4435474-supplementary.pdf]

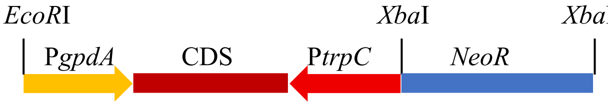

Figure S1. Construction of *IscA1* gene RNA interference vector. The RNA interference cassette and *NeoR* fragment were ligated into pCAMBIA3300 plasmid to construct the pCAMBIA-RNAi-R vector.

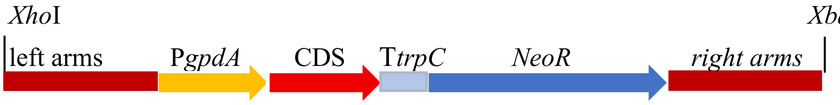

Figure S2. Construction of *IscA1* gene overexpression vector. The six DNA fragments were merged into a single larger DNA fragment, which was subsequently ligated into the pCAMBIA3300 plasmid to create the *IscA1* overexpression vector.

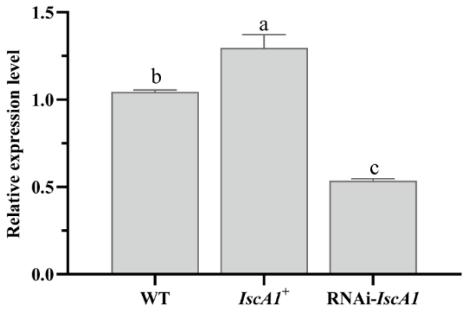

Figure S3. The relative expression levels of *IscA1* gene in the *IscA1*$^{+}$ and RNAi-*IscA1* strains. Note: WT, wild type; *IscA1*$^{+}$, *IscA1* overexpression strain; RNAi-*IscA1*, RNA interference strain. Different lowercase letters above the columns of the same gene indicate significant differences (p<0.05).

Table S1 Primers for constructing the *IscA1* gene RNA interference vector

Primer names	Primer sequences (5'-3')	Functions
P*gpdA*F*EcoR1*	GAATTCGCATGCGGAGAGACGGACGG	P*gpdA* *amplification*
P*gpdA*R	TGGGAAAAGAAAGAGAAAAG	
*IscA1*F	CTTTTCTCTTTCTTTTCCCACGGAATTCATGTCGTTTTGC TGCACCGT	CDS amplification
*IscA1*R	CGAAGCTAATCCTTCATTGGTCAAACCATGAAAGATT	
P*trpC*F	CGGGATCCTCGACAGAAGATGATATTGA	P*trpC* amplification
P*trpC*R	GGTTACTTCCTAATCGAAGC	

Table S2 Primers for constructing the *IscA1* gene overexpression vector

Primer names	Primer sequences (5'-3')	source
*IscA1*UPF*XhoI*	CCGCTCGAGTTTTAGGAGCGTTATCCAG	5'UTR amplification
*IscA1*UPR	TCCGTCTCTCCGCATGCTGGAGGTGTCT GGGATAG	
P*gpdA*F	GCATGCGGAGAGACGGACGG	P*gpdA amplification*
P*gpdA*R*EcoR1*	GAATTCTGGGAAAAGAAAGAGAAAAG	
*IscA1*F*EcoR1*	CGGAATTCATGTCGTTTTGCTGCACCGT	CDS amplification
*IscA1*R*BamHI*	CGGGATCCTCAAACCATGAAAGATT	
T*trpC*F*BamHI*	CGGGATCCGATCCACTTAACGTTACTGA AAT	T*trpC* amplification
T*trpC*R	GTTCGATGGGGTTGAGTTGGTGCATTGC AGATGAGCTGTAT	
G418F	CCAACTCAACCCCATCGAACCGTAACCC CAT	Amplification of *Neo* resistance genes
G418R	ACATGCATGTTGCATGATGAT	
*IscA1*DWF	CAGTACATGCATGTTGCATGATGATTGA GAAAGGGGCGGCTGGTCCAT	3'UTR amplification
*IscA1*DWR *XbaI*	GCTCTAGACACTCAGCTCATCTTCTGAA AGGAT	
INF	GAGCTTAATAGAAGGGACGGT	Fixed-point conversion sub validation
INR	CCGTATGTCACTCGATCCATTCT	

Table S3 Primers of genes used for real-time quantitative PCR

Primer names	Primer sequences (5'-3')	Amplification product	The corresponding protein ID in *Monascus purpureus* in JGI database
RT*IscA*F	ACAGCTTCGCAAGCTACTATC	108bp	501839
RT*IscA*R	GGCTTCGCCACGTATTCAAG		
RT*IscS*F	GCAACAACTCCGCTTGATCC	113bp	498245
RT*IscS*R	GCCTTCTCCGATTCCCATCC		
RT*IscU*F	CTGCATGCGGTGATGTGATG	112bp	449506
RT*IscU*R	CTAGAGCTAGCAATGGCGCT		
RT*GrxD5*F	GGTGTAGACCCCAAGAAGTT	94bp	501570
RT*GrxD5*R	GTATTGTTGGCCAATCCGAG		
RT*HapX*F	GACACCACCTGGTTTGTTAC	70bp	120137
RT*HapX*R	CGACAATTATTTGGGGGCTG		
RT*SreA*F	ACCTCCTTCTTGGGACATTC	108bp	521524
RT*SreA*R	CAGATAGAGACCACAAGCGT		
RT*pksPT*F	GGCAACCTTCAGTCCGCTAT	162bp	512724
RT*pksPT*R	GATCAGTGCGATGCCATGTG		
RT*pigR*F	ACTCTGGAAAGCTGCTTCGG	122bp	504641
RT*pigR*R	GGACGTTCTGGATGGCGTAT		
RT*pksCT*F	AACCATGGAGGCATTGGACC	235bp	509802
RT*pksCT*R	CCTTGTCGGTCACACCGAAT		
RT*ctnR*F	AGTATCTGGATGGACGGGTG	140bp	444645
RT*ctnR*R	ACGACGACGAGTGTCAGTTC		
*GAPDH*F	GTCTATGCGTGTGCCTACTTCC	156bp	499003
*GAPDH*R	CGTGTGCAACATATCAGCGTC		
